# Supplementary material for: MeJA Elicitation of Chicory Hairy Roots Promotes Efficient Increase of 3,5-diCQA Accumulation, a Potent Antioxidant and Antibacterial Molecule
Source: Antibiotics (Basel). 2020 Sep 30;9(10):659. doi: 10.3390/antibiotics9100659 (PMC7601367; doi:10.3390/antibiotics9100659)

**Supplementary Figure S1 .** Analytical HPLC chromatogram (A) and preparative HPLC chromatogram (B) of ethyl acetate sub-extract from hairy root elicitated with MeJA: (1) di-CQA; (2) tri-CQA.

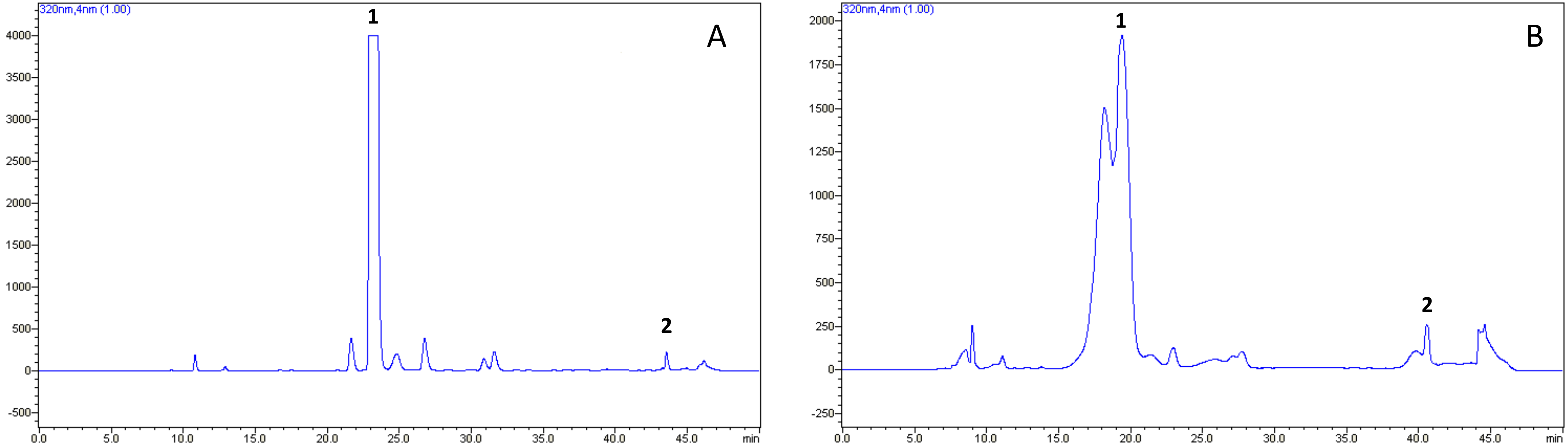

Supplement: Supplementary file 1 [file antibiotics-09-00659-s001.zip › Supplementary Figure S1.pdf]
