# Supplementary material for: MeJA Elicitation of Chicory Hairy Roots Promotes Efficient Increase of 3,5-diCQA Accumulation, a Potent Antioxidant and Antibacterial Molecule
Source: Antibiotics (Basel). 2020 Sep 30;9(10):659. doi: 10.3390/antibiotics9100659 (PMC7601367; doi:10.3390/antibiotics9100659)

Supplementary Figure S2. Chromatograms at 320 nm of the crude methanolic extract of HR2. (A) Aqueous sub-extract of HR2. (B) Ethyl-acetate sub-extract of HR2. (C) Pure compounds (1) 3-caffeoylquinic acid (CQA); (2) 3,5-dicaffeoylquinic acid (di-CQA); (3) 3,4,5-tricaffeoylquinic acid (tri-CQA)

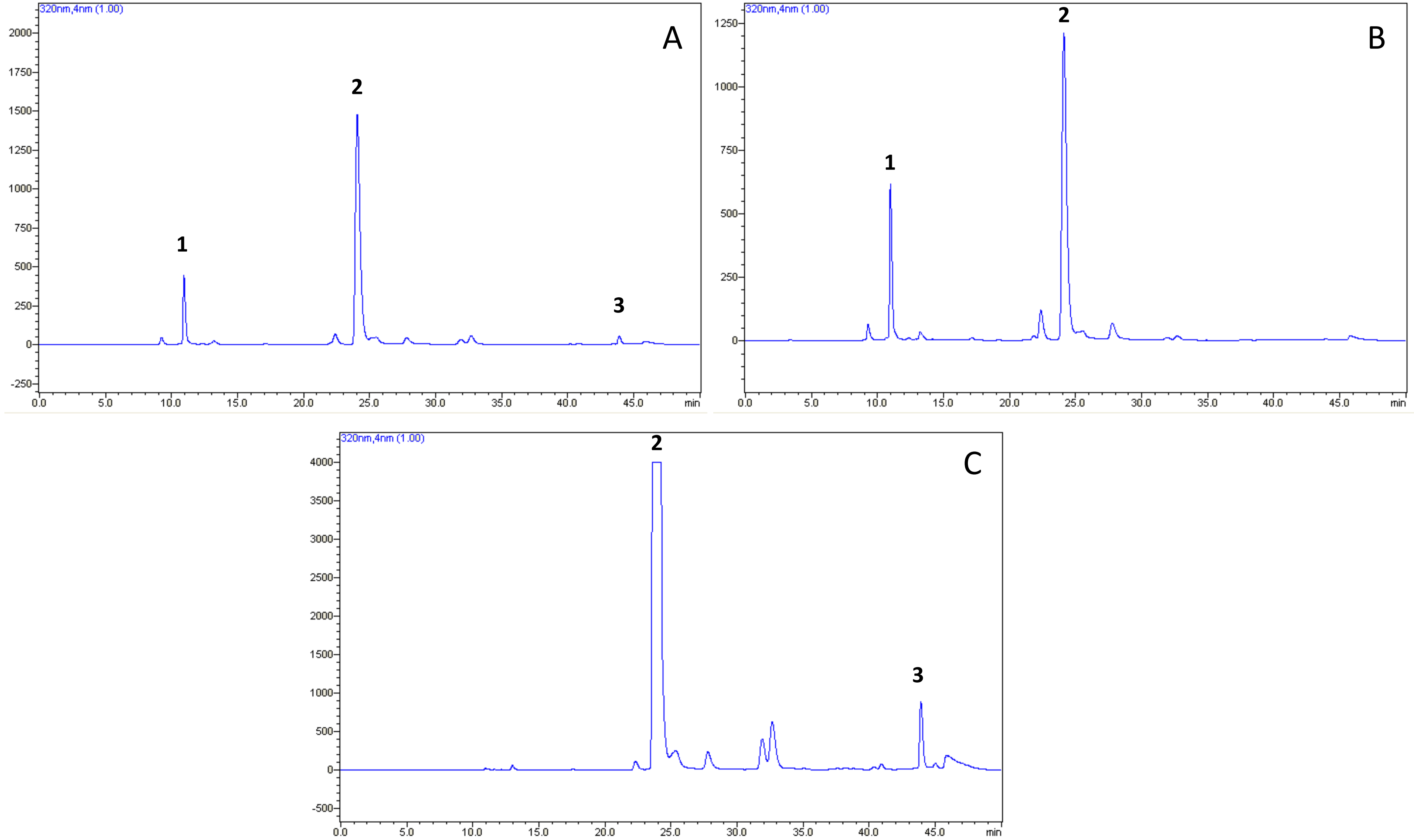

Supplement: Supplementary file 1 [file antibiotics-09-00659-s001.zip › Supplementary Figure S2.pdf]
